# Evaluating markers in selected genes for association with functional longevity of dairy cattle

**DOI:** 10.1186/1471-2156-12-30

**Published:** 2011-03-10

**Authors:** Joanna Szyda, Małgorzata Morek-Kopeć, Jolanta Komisarek, Andrzej Żarnecki

**Affiliations:** 1 Department of Animal Genetics, Wrocław University of Environmental and Life Sciences, Kożuchowska 7, 51-631 Wrocław, Poland; 2 Institute of Natural Sciences, Wrocław University of Environmental and Life Sciences, Norwida 25, 50-375 Wrocław, Poland; 3 Department of Genetics and Animal Breeding, University of Agriculture in Cracow, Mickiewicza 24/28, 30-059 Cracow, Poland; 4 Department of Cattle Breeding and Milk Production, Poznan University of Life Sciences, Wojska Polskiego 71A, 60-625 Poznań, Poland; 5 National Research Institute of Animal Production, Balice, 32-083 Cracow, Poland

## Abstract

**Background:**

Longevity expressed as the number of days between birth and death is a trait of great importance for both human and animal populations. In our analysis we use dairy cattle to demonstrate how the association of Single Nucleotide Polymorphisms (SNPs) located within selected genes with longevity can be modeled. Such an approach can be extended to any genotyped population with time to endpoint information available. Our study is focused on selected genes in order to answer the question whether genes, known to be involved into the physiological determination of milk production, also influence individual's survival.

**Results:**

Generally, the highest risk differences among animals with different genotypes are observed for polymorphisms located within the leptin gene. The polymorphism with a highest effect on functional longevity is LEP-R25C, for which the relative risk of culling for cows with genotype CC is 3.14 times higher than for the heterozygous animals. Apart from LEP-R25C, also FF homozygotes at the LEP-Y7F substitution attribute 3.64 times higher risk of culling than the YY homozygotes and VV homozygotes at LEP-A80V have 1.83 times higher risk of culling than AA homozygotes. Differences in risks between genotypes of polymorphisms within the other genes (the butyrophilin subfamily 1 member A1 gene, BTN1A1; the acyl-CoA:diacylglycerol acyltransferase 1 gene, DGAT1; the leptin receptor gene, LEPR; the ATP-binding cassette sub-family G member 2, ABCG2) are much smaller.

**Conclusions:**

Our results indicate association between LEP and longevity and are very well supported by results of other studies related to dairy cattle. In view of the growing importance of functional traits in dairy cattle, LEP polymorphisms should be considered as markers supporting selection decisions. Furthermore, since the relationship between both LEP polymorphism and its protein product with longevity in humans is well documented, with our result we were able to demonstrate that livestock with its detailed records of family structure, genetic, and environmental factors as well as extensive trait recording can be a good model organism for research aspects related to humans.

## Background

The major goal of our study was to evaluate the association between SNP markers located within selected genes and functional longevity of dairy cows. For this purpose effects of SNPs within genes representing different groups regarding their effect on milk production were selected: (i) genes with well characterised high effects on milk production (two genes), (ii) genes which effect on milk production is contradictory, depending on the analysed data set (two genes), (iii) genes which effects on milk production have not been reported (one gene).

Longevity expressed as the number of days between birth and death is a trait of great importance for both human and animal populations. In our analysis we use dairy cattle as an organism for which we demonstrate how the influence of particular genes on longevity can be modelled. Such an approach can be extended to any genotyped population with time to endpoint information available. On one hand dairy cattle makes a good model organism since it has precise current and historical pedigree data as well as information on herd management, various traits, medical examinations etc. recorded through individuals lifetime. On the other hand however, the peculiarity of the survival analysis of dairy cattle data is that in this case the endpoint (i.e. culling) is not only dependent on individuals' health status, but involves many factors like individual's production level or economic issues. Consequently, in dairy cattle breeding one can differentiate between true longevity - where the actual probability of animal's survival is considered and functional longevity - where additional effects expressing the risk of culling a cow irrespective of her production performance level and herd management are included into the modelling of the probability of survival [1].

Functional longevity is rapidly gaining importance as a selection criterion in dairy cattle. Therefore many studies have analyzed its relationship with other characters routinely recorded in dairy cattle, such as reproduction traits [2], udder health traits [3], and level of inbreeding [4] etc. However, from a geneticist's perspective knowledge whether there exist genes which influence functional longevity is of primary importance.

The genetic effect on survival has been considered in various aspects by other authors. Probably the most widespread approach is to model a cumulated additive effect of all genes, called a polygenic effect, which is routinely estimated for sires of cows from production populations in many countries [5]. Diao *et al. *[6], Diao and Lin [7], Moreno *et al. *[8] and Johannes [9] estimated position and effect of quantitative trait loci using linkage analysis applied to simulated and real data. Tregouet *et al. *[10] and Souverein *et al. *[11] estimated SNP haplotype effects on failure time for humans. Our study is focused on particular candidate genes in order to answer the question whether selected genes, known to be involved into the physiological determination of milk production, also influence individual's survival.

## Results

**Figure 1:**
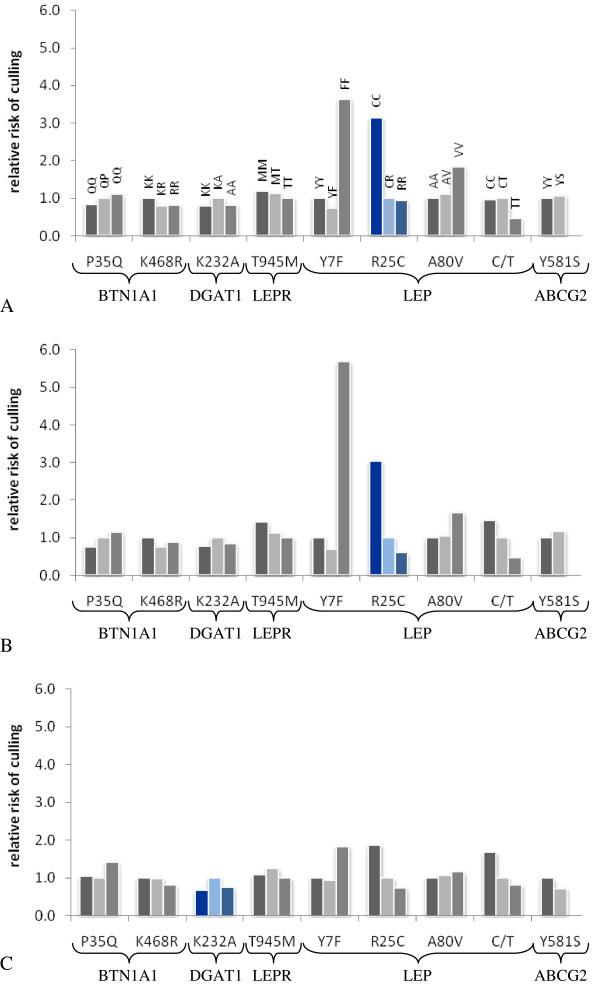
**Relative risk of culling for different genotypes estimated based on model M_20_**. Polymorphisms with the lowest FDR based on the comparison of M_20 _with (M_11_-M_19_) are marked in blue. A. Weibull model for functional longevity. B. Cox model for functional longevity. C Weibull model for true longevity.

All comparisons of the most parsimonious model M_1 _with nine other models including a single polymorphism (M_2_-M_10_) as well as comparisons of the full model M_20 _with the nine models with one polymorphism excluded (M_11_-M_19_) remain in agreement, showing that the polymorphism with a highest effect on functional longevity is LEP-R25C, located within the leptin gene (Table 1). In particular, including the effect of LEP-R25C into the model resulted in the improvement of fit over the basic model with FDR = 0.2574, corresponding to the nominal P value of 0.0286, while excluding the effect of this polymorphism from the full model resulted in a decreased fit with FDR = 0.1863, corresponding to the nominal P value of 0.0207. Relative risk of culling for cows with genotype CC is much higher than for individuals with the remaining two genotypes. Based on M_20_, it is 3.14 times higher than for the heterozygous animals. Generally, the highest risk differences among animals with different genotypes are observed for polymorphisms located within the leptin gene. Apart from the abovementioned LEP-R25C, also FF homozygotes at the LEP-Y7F substitution attribute 3.64 times higher risk of culling than the YY homozygotes (the estimate of risk for FF at Y7F is based on a single individual). VV homozygotes at LEP-A80V have 1.83 times higher risk of culling than AA homozygotes. Differences in risks between different genotypes of polymorphisms within the other genes are much smaller (Figure 1A).

**Table 1:** FDR values of the likelihood ratio test (λ) for comparisons of different models.

model description	BTN1A1		DGAT1	LEPR	LEP				ABCG2
	
	P35Q	K468R	K232A	T945M	Y7F	R25C	A80V	C/T	Y581S
	no SNP vs. single SNP								
functional longevity, Weibull	0.8377	0.9374	0.3591	0.8377	0.3911	**0.2574**	0.3591	0.3591	0.8377

	one SNP excluded vs. all SNPs								
functional longevity, Weibull	0.5954	0.4413	0.4307	0.7959	0.4307	**0.1863**	0.2372	0.4307	0.8540
functional longevity, Cox	0.2423	0.2423	0.2665	0.6899	0.2423	**0.2151**	0.2423	0.2423	0.6929
true longevity, Weibull	0.6404	0.9006	**0.1341**	0.6829	0.9006	0.6829	0.9006	0.9006	0.7160

The lowest value is given in bold.

Using either an unspecified baseline hazard function underlying the Cox model, or a specified Weibull baseline hazard function, did not affect the estimates of polymorphisms markedly. LEP-R25C was the polymorphism with the largest effect on survival (Table 1; Figure 1A, 1B). On the other hand differences arise between a functional and a true longevity model. As far as true longevity is concerned, the most significant polymorphism is DGAT1-K232A (FDR = 0.1341, nominal P = 0.0149) with the highest risk of culling corresponding to heterozygous cows (Figure 1C).

Other (than SNP) effects with significant impacts on functional survival were estimated for protein yield level, lactation number × stage of lactation, and year × season. For those effects risk of culling in first lactation was higher than in subsequent lactations, within lactation always most prone to culling were cow in last stage, high risk was associated with low protein production level. True longevity was also significantly affected by lactation number × stage of lactation, and year × season effects.

## Discussion

The advantage of using a survival analysis model, which takes into account data censoring, was demonstrated by Moreno *et al. *[8]. Using simulated data, the authors showed that for data sets containing censored observations power of quantitative trait loci detection and accuracy of the estimation of its position and effect is higher for survival models with Weibull and Cox hazard functions than for a standard QTL analysis model, which ignores censoring. The adequacy of modeling gene effects on survival data was also shown in the analysis of real data by Diao *et al. *[6]. Performing survival linkage analysis for time to death after bacterial infection of mice using a Weibull hazard function the authors were able to confirm previously identified quantitative trait loci as well as to found a novel locus.

Our study concerned the association of functional longevity with SNPs located within selected genes and found some, albeit not strong, effect of LEP. Although there exist studies which have found significant association of polymorphisms identified within the leptin gene with production traits (e.g. [12]), relation between LEP polymorphism and fitness, metabolism or health related traits, which may be components of functional longevity, are much less common and their results are contradictory. Banos *et al. *[12] failed to observe a significant association of LEP with body energy traits, but Liefers *et al. *[13] and Nkrumah *et al. *[14] reported significant effects of LEP on growth rate, live weight, metabolic body weight, feed intake, and feeding duration. Chebel *et al. *[15] observed significant associations of LEP with disease risk. Oikonomou *et al. *[16] detected significant effects of LEP on body energy and blood metabolic traits. Brickell *et al. *[17] reported significant associations between polymorphisms in the leptin gene with stillbirths and calf mortality. Anyhow, the involvement of leptin gene in inflammation and immune responses [18] makes it a good candidate for a genetic component of longevity.

Although it could have been hypothesized that genes with high effect on yield may also influence longevity, which could be due to redirecting body energy resources towards milk, fat or protein production, no evidence for such phenomenon was observed in our study. The effects of polymorphism located within genes of high impact on milk performance (DGAT1, ABCG2) were not different from a gene with no effect documented (BTN1A1).

## Conclusions

As it could have been expected, when information on cows' production level is ignored in the model corresponding to a true longevity, the most significant effect is attributed to DGAT1 - a polymorphism well known to be responsible for milk production traits in dairy cattle [19]. Cows with a low production level are then culled earlier than their high producing herdmates.

Our results indicate an association between LEP and functional longevity. As such functional longevity is a complex trait, involving various physiological components acting throughout a cow's production life. On one hand our results are very well supported by other studies involving traits like health, feeding behavior, or reproduction, which are components of functional longevity. On the other hand, the reliability of results obtained is hampered by a moderate size of the data set. Still, in view of the growing importance of functional traits in dairy cattle, LEP polymorphisms should be considered as markers supporting selection decisions.

The relationship between both LEP polymorphism and its protein product with longevity in humans has been documented [20,21] indicating the impact of the gene on reduction in visceral fat, blood glucose, and insulin levels together with increased concentration of ghrelin [22]. With our results we were able to demonstrate that livestock with its detailed records of family structure, genetic, and environmental factors as well as extensive trait recording can be a good model organism for research aspects related to humans.

## Methods

### DNA samples

DNA samples were extracted from 566 Polish Holstein-Friesian cows for which genotypes at 9 functional single nucleotide polymorphisms (SNP) located within 5 genes were identified:

a) the butyrophilin subfamily 1 member A1 gene (BTN1A1, GeneID:282157) located on BTA23: P35Q [23] and K468R [24];

b) the acyl-CoA:diacylglycerol acyltransferase 1 gene (DGAT1, GeneID:282609) on BTA14: K232A [19; 25];

c) the leptin receptor gene (LEPR, GeneID:497205) on BTA3: T945M [26];

d) the leptin gene (LEP, GeneID:280836) on BTA4: Y7F [27], R25C and A80V [28], a C/T substitution at position -963 [29];

e) the ATP-binding cassette sub-family G member 2 gene (ABCG2, GeneID:536203) on BTA6: Y581S [30].

Regarding the effects of the selected genes on milk production traits - i.e. traits posing the major artificial selection pressure in dairy cattle populations, DGAT1 and ABCG2 are known to affect milk production traits, for LEP and LEPR contradictory results have been obtained, while the effect of BTN1A1 has not been reported. Genotypes were determined using PCR-RFLP method, as described by Szyda and Komisarek [31] for BTN1A1, DGAT1, LEP, LEPR and by Komisarek and Dorynek [32] for ABCG2.

### Data structure

The analyzed data consisted of 567 genotyped cows, daughters of 109 sires. No missing genotypes were present in the analysed sample, but for ABCG2-Y581S and Y2F, with 0 and 1 cows respectively, the genotype class counts were strongly skewed Table 2. Minor allele frequency varied between 0.013 for ABCG2-Y581S and 0.481 for LEP-R25C. The average number of daughters per sire was 5 and ranged from 1 to 67. The cows had their production records in four herds. The distribution of cows among herds was not uniform, with 80% of cows (453) active in the same herd. 65% (368) of the cows were represented by individuals with observed culling dates (i.e. uncensored records), with the average failure time of 1 173 days, varying between 32 and 3 168 days. The remainder of the cows comprised animals with so called censored records, represented by cows which have not been culled within the data collection timeframe. For them the censoring times varied between 292nd and 3 046th day, with the average censoring time amounting to 1489 days. For this data the following classes, corresponding to the Polish national routine genetic evaluation model for functional longevity were considered: year × season - comprising years 1999 to 2009 and 2 seasons: April - September and October - March; lactation number × stage of lactation - comprising the first 5 and pooled later lactations and 4 stages of lactation (1-29, 30-179, 180-304, and >304 day of lactation); relative change of herd size from the current year to the next year at April 1st (<-50%, -50% to -30%, -30% to -10%, -10% to 10%, 10% to 30%, 30% to 50%, > 50%); classes of 305-day fat and protein yield levels relative to herd means, defined separately for the first and later lactations (<-50%, -50% to -40%, -40% to -30%, -30% to -20%, -20% to -10%, -10% to 0%, 0% to 10%, 10% to 20%, 20% to 30%, 30% to 40%, 40% to 50%, > 50%); monthly classes of age at first calving (≤20, 21, 22,..., >40 month); 3 classes of SNP genotypes represented by two homozygotes and a heterozygote.

**Table 2:** Number of cows in each genotype class

BTN1A1		DGAT1	LEPR	LEP				ABCG2
P35Q	K468R	K232A	T945M	Y7F	R25C	A80V	C/T	Y581S
QQ = 168	KK = 411	KK = 80	MM = 5	YY = 523	CC = 133	AA = 283	CC = 157	YY = 552
QP = 280	KR = 146	KA = 282	MT = 106	YF = 43	CR = 280	AV = 231	CT = 287	YS = 15
PP = 119	RR = 10	AA = 205	TT = 456	FF = 1	RR = 154	VV = 53	TT = 123	SS = 0

### Statistical model

The analysed trait is defined as the number of days between the first calving and culling (uncensored records) or the last test day (censored records). The following sequence of functional longevity survival models based on Weibull hazard function was applied to the data:

M_1_:

M_2_-M_10_:

M_11_-M_19_:

M_20_:

where *h_0_(t) = ρ(t)^ρ-1^exp[ρ log(λ)] *represents a baseline Weibull hazard function with scale parameter λ and shape parameter *ρ, ys(t) *is a time-dependent fixed effect of year-season, *sl(t) *is a time-dependent effect of lactation number × stage of lactation, *hsize(t) *is a time-dependent fixed effect of yearly herd size variation, *fat(t) *and *prot(t) *are time-dependent fixed effects of within herd-year-season classes of 305-day fat and protein production level, *age *is a time-independent fixed effect of age at first calving, *SNP*_i _represents a time independent additive effect of a single polymorphism *i *(*i *∈ {P35Q, K468R, K232A, T945M, Y7F, R25C, A80V, a C/T substitution at position -963, and Y581S}), *SNP-*_i _represents a time independent additive effect of eight of the polymorphisms excluding *i*. For comparison, the effects underlying M_20 _were also estimated using a Cox model in which *h_0_(t) *is not expressed by a predefined function what corresponds to making no assumption on the underlying baseline risk. Note, that all the above models represent functional longevity models, which aim to estimate effects of SNPs on culling risk irrespectively of cows' productive performance. Since only animals not culled for milk production contribute meaningful information on longevity, the effects of production level (fat and protein yield class effects) were included into the model in order to make estimates of SNP effects independent from production level. Additionally a modification of M_20 _with effects of fat and protein production level removed, corresponding to a true longevity was applied to the data. The estimation of model parameters was performed using Survival Kit Version 3.12 [33].

### Hypotheses testing

The hypothesis of interest was whether some of the considered polymorphisms influence the risk of cow's culling and was tested using the likelihood ratio test: , where  and  represent maximum of likelihood functions obtained under the more parsimonious and the less parsimonious model, respectively. In this analysis model parsimony is expressed by the number of polymorphisms considered, while the other model parameters remain the same for all the models. Asymptotically, λ follows the χ^2 ^distribution with the degrees of freedom equal to the difference in the number of effects in the compared models.

All experiments were carried out in compliance with ethical requirements and were approved by the Local Ethics Committee for Animal Research (permission No. 25/2003).

## Authors' contributions

JS provided the idea for the study, as involved in statistical analysis of the data and wrote the manuscript. MMK edited data and performed all computations. JK collected DNA samples and provided genotypes. AZ provided intellectual support and helped in manuscript preparation. All authors read and approved the manuscript.
